# Molecules at Play in Cancer

**DOI:** 10.3390/cimb45030140

**Published:** 2023-03-07

**Authors:** Dumitru Andrei Iacobas

**Affiliations:** Personalized Genomics Laboratory, Undergraduate Medical Academy, Prairie View A&M University, Prairie View, TX 77446, USA; daiacobas@pvamu.edu

Despite its wide range of incidence, cancer can spontaneously occur in any part of the body and invade regions other than the originally affected tissue. Extensive research conducted by thousands academic institutions all over the world and huge industry investments in developing diagnostic bioassays and therapeutic agents spanning decades are yet to produce valuable results for a better understanding of cancer formation and the design of effective treatments. From genes to transcripts, proteins (enzymes included) and metabolites, a long list of molecular factors are believed to be responsible for triggering the transformation of normal cells into cancer cells, many of these factors being also considered as potential actionable molecules for targeted therapies. 

For these reasons, the most significant recent studies on molecular-based diagnostics and the molecular therapy of several types of cancer are collated in this Special Issue of *CIMB*, entitled “Molecules at Play in Cancer”. The 24 chapters present the contributions of 166 researchers from 16 countries: Brazil (14), China (12), Ecuador (2), Greece (10), Hungary (8), Italy (4), Japan (25), Korea (29), Mexico (11), Poland (5), Portugal (2), Russia (2), Switzerland (7), the UK (1) and the USA (27). The articles reported new molecular findings regarding the following types of cancer: blood [1,2], breast [3,4,5], kidney [6], liver [7,8], lung [9,10,11,12], ovary [10,13,14], prostate [15], soft tissue [16], gastric [17,18], testicular [19], cervical [20,21], and adrenal gland [22]. 

However, beyond the relevance of these studies for understanding the roles of various molecular factors in cancer development and anti-cancer therapies, I would like to discuss the types of investigated biological specimens that provided experimental evidence for the authors’ conclusions. The types of the specimens were: (1) fresh tissues from cancer patients and healthy counterparts; (2) formalin-fixed, paraffin-embedded (FFPE), long-term-stored human tissues; (3) standard and genetically engineered human cancer cell lines; (4) tissues from diseased and healthy animal models; (5) cancer nodules and surrounding normal tissue from the same tumor of each patient.

Due to large numbers, comparing either fresh or FFPE samples from cancer and healthy humans has the advantage of statistical significance, especially when the investigated populations are homogeneous regarding race, sex, age group and other important cancer-favoring factors. When profiled in-house, experiments on human tissues require the approval of the local Institutional Review Board (IRB), which often limits the spectrum of the experimental approaches. The use of either cell lines or animal models, even with the required Institutional Animal Care and Use Committee (IACUC) approval, has the advantage of allowing the genetic engineering and testing of various treatments.

Most authors of this Special Issue compared fresh tissue samples from cancer patients with those obtained from healthy counterparts in order to identify novel biomarkers or simply test the predictive value of already established biomarkers. Thus, basigin, a membrane-bound glycoprotein, was identified by Łacina et al. [2] as a multiple myeloma biomarker by comparing the expression of the encoding gene in the peripheral blood from 62 patients with multiple myeloma with that of 25 healthy donors. The studied cancer patients were sometimes subdivided into groups based on the etiology of their disease. For instance, Armakolas et al. [8] compared the proteome profiles, expression of selected miRNAs and abundance of circulating tumor cells among a group of 56 people with advanced and 33 people with early hepatocellular carcinoma, 28 with cirrhosis and 5 healthy controls. Barbirou et al. sequenced circulating tumor cells to identify somatic variants in people with non-small-cell lung cancer [9]. An interesting study by Bel’skaya et al. [5] compared the saliva “omic” characteristics of 487 females with breast cancer with those of 298 healthy controls. Seipel et al. proved that anti B-cell maturation antigen CAR-T cell treatment is efficient in four out of five cases of patients with relapsed multiple myeloma [1].

The significance of infection with human papillomavirus (HPV) in the development of breast cancer was tested by Maldonado-Rodríguez et al. [4] through quantifying the presence of the HPV DNA in 116 formalin-fixed breast tissues of 59 malignant neoplasms, 5 in situ neoplasms, 1 borderline neoplasm and 20 benign neoplasms. The study concluded that virus presence is not a sufficient condition for developing cancer. However, as shown by Hayashi et al. [10], the mortality of infectious diseases such as COVID-19 is higher for women with a pulmonary metastatic niche caused by ovarian adenocarcinoma.

Both fresh and formalin-fixed tissues were used by Go et al. [18] to assess the association of a high expression of epidermal growth factor receptor and cyclin D1 with gastric cancer, and by Jeon et al. [14] to test the association of circulating exosomal miR-1290 with epithelial ovarian cancer. This strategy increases the statistical relevance of studies, while providing a direct evaluation of how much formalin fixation and long-term storage affects the expression levels of the biomarkers.

Other authors used human cancer cell lines to elucidate the responsible molecular mechanisms. Thus, HeLa cells were used by Zhang et al. [21] to test the role of Parkin as synergistic mediator of mitophagy in dysfunctional mitochondria. The testicular teratocarcinoma NCCIT and NTERA2 lines helped Chen et al. [19] to evaluate the therapeutic role of PNU-74654, and prostate cancer LNCaP and DU145 lines were used by Iacobas and Iacobas [15] to identify gene master regulators.

For several studies, genomic data were downloaded from The Cancer Genome Atlas (TCGA). Liu et al. [17] used these data to determine the prognostic value of miR-942-3p for the gastric cancer, Ferreira et al. [6] to determine the value of *GOT2* in clear–cell renal-cell carcinoma, and Kim et al. [13] to determine that of zinc finger E-box binding homeobox 2 in ovarian cancer.

Although most of these studies were carried out on human samples, two groups, Somlyai et al. [12] and Gutierez et al. [16], used mouse models, while Almeida et al. [23] tested the proposed solutions on bacteria. A comprehensive review by Lucio et al. [24] presents the major accomplishments in cancer research that have been possible by the use of the nematode *Caenorhaditis elegans*.

The interesting articles of this Special Issue reveal the potential anti-cancer benefits of using deuterium-depleted water (Somlyai et al., [12]) or even the broad-spectrum antiparasitic activity of the dog dewormer Fenbendazole (Sultana et al. [11]), which is sometimes self-administrated by desperate Korean patients. 

Two articles indicated that race and sex should be considered when discussing the relevance of cancer biomarkers. Thus, an epidemiological study by Andrade et al. [20] identified the genetic heterogeneity among subpopulations with different ancestry in Brazil, and sex differences were analyzed in a mouse model of embryonal rhabdomyosarcoma by Gutierez et al. [16].

Nevertheless, in addition to race, sex and age, combinations of cancer risk factors such as medical history, diet, habits and exposure to stress, toxins and radiation make each human a dynamic unique subject. Therefore, is it really possible to identify biomarkers characterizing all patients with a particular form of cancer? Although preferred by the pharma industry for economic reasons, are “fit-for-all” treatments really effective for everyone? Tumor heterogeneity further complicates the characterization of cancer subtypes and requires complex approaches to destroy most of the primary cancer clones at once. Moreover, both the uniqueness of favoring factors for each person and tumor heterogeneity question the validity of meta-analyses that compare the genomes and/or transcriptomes of subpopulations of cancer-stricken and healthy individuals. Instead, we should refer the genomes and/or transcriptomes of cancer cells to those of the normal cells within the tissue of the same person, with an emphasis on how to better personalize treatment to fit patient own characteristics [15].
